# AI finally provides augmented intelligence to liver surgeons

**DOI:** 10.1016/j.ebiom.2020.103064

**Published:** 2020-10-20

**Authors:** Nicolas Golse

**Affiliations:** 1 Department of Surgery, Paul-Brousse Hospital, Assistance Publique des Hôpitaux de Paris, Centre Hépato-Biliaire, Villejuif, 94800, France; 2 Université Paris-Saclay, INSERM, Physiopathogénèse et Traitement des Maladies du Foie, UMR-S 1193 France

In their paper untitled “Deep Learning Quantification of Percent Steatosis in Donor Liver Biopsy Frozen Sections » [1], L. Sun et al. propose a new method to quantify graft macrosteatosis (MS) during liver procurement. Their technology is based upon a deep learning convolutional neural network that generates a steatosis probability map from an input whole slide image of a hematoxylin and eosin-stained frozen section, and subsequently calculates the percentage of steatosis. The learning was performed on a training set (*n*=30) and then applied on a second input test set (*n*=66). Deep learning models were superior to the estimates of the on-service pathologists at the time of initial evaluation (frozen section) and well correlated with definitive pathological quantifications.

This study answers to a real need as every liver surgeon knows that MS is a major predictor of early graft dysfunction and that macroscopic evaluation poorly correlates with MS content. Despite its monocentric design and small sample size, this work represents a potential breakthrough in the field of liver transplantation, as we were, so far, facing a paradox: pathological evaluation is still considered as the gold standard way to assess the MS content of grafts while it has been reported many times that frozen section analysis was not reliable [2] and that there was a huge inter/intra-operator variability [3]. For these reasons, we really needed a reproducible and accurate tool for MS quantification and that may be what this US team is proposing here. Moreover, many software performing automatic calculation of MS content are already available, but none (or almost none) allows such estimation from frozen section slides, thus limiting their clinical utility. The present work clearly represents a significant advance for clinical acceptance and daily use of this equipment, regardless of the time of the day. The solution proposed by L. Sun et al. is a concrete and practical example of multidisciplinary collaboration (clinicians, computer scientists, biostatisticians, mathematicians) to improve healthcare performance without the need for major modifications in the current logistic organization of procurements. This automatic process provides better results than human analyses performed on frozen section specimens, and it could lead to improve the results of liver transplantation, preventing unnecessary organs discard or steatotic grafts acceptance. The specific focus on MS provided by their technique definitively represents a major asset for clinical decision as there is a global consensus toward a greater impact of MS than microsteatosis. Moreover, in the near future, this deep learning application could be hosted online, with the assessment performed via cloud computing: the user would upload the slide, run the assessment and receive the MS quantification within a few minutes. This could allow a wide dissemination of the technique, regardless of the local level of expertise and human resources. In addition to the current use proposed, deep learning MS quantification could be advantageously used repeatedly during the defatting therapies of highly-steatotic machine-infused organs [4].

The main concern raised by this workflow is that a pathologist (or technician) is still necessary to prepare the slide and run the software. In European centers where procurements are performed, a pathologist is not always available at night in every peripheral hospital. That is why we still consider other alternatives, as the one we recently reported [5], as useful as those proposed by L. Sun et al.

Surprisingly, new digital technologies are more and more present in our daily life, but they still don't have impregnated so much the medical area, and particularly decisional-making process. Excessive expectations from Artificial Intelligence (AI) led to disappointment and disillusionment in healthcare [6]. Some successful experiences combining AI and clinical decision-making have been recently reported [7] and, obviously, automated medical-image diagnosis is the most successful domain of AI applications, as well as dermatology, ophthalmology and genome interpretation [8]. The use of AI for preoperative planning and intraoperative guidance, associated with its integration into surgical robots, will probably increase the surgeon's acceptance of these new technologies. Let us not lose sight of the fact that the resolution of the main technical problems raised by AI must now encourage us to resolve major new challenges, in particular those posed by privacy, ethical and legal issues [9]. As an example, who will be responsible in case of wrong choice of a graft that caused a primary non function or recipient's death? More than ever, technology, even fully automatic and totally robust, puts the clinician at the heart of the decision-making process. It provides a long-awaited solution to human issues (reproducibility and availability of pathologists in the middle of the night) and technical problems (artefact in case of extemporaneous analysis), but cannot and should not erase humans (humanism?) from the chessboard. The final decision must remain multifactorial and integrate a set of factors still impossible to model. Finally, AI allows the clinicians to have an augmented intelligence, superior to an empirical (or even *evidence based*) human decision. This change in the way of approaching medical problems and of proposing solutions will necessarily have to be rigorously evaluated to ensure that it provides clear added value, reaching the expected clinical, economic and / or educational benefits. This assessment, as well as external validation of these preliminary results, are eagerly expected from L. Sun et al. . Performance comparison with already well-established automatic MS quantification techniques would be also beneficial to promote the use avec Deep Learning assessment [10].

## Declaration of Competing Interest

Nothing to disclose.
